# Characterisation of *S. aureus*/MRSA CC1153 and review of mobile genetic elements carrying the fusidic acid resistance gene *fusC*

**DOI:** 10.1038/s41598-021-86273-4

**Published:** 2021-04-14

**Authors:** Stefan Monecke, Elke Müller, Sascha D. Braun, Marc Armengol-Porta, Michèle Bes, Samar Boswihi, Maged El-Ashker, Ines Engelmann, Darius Gawlik, Mayada Gwida, Helmut Hotzel, Rania Nassar, Annett Reissig, Antje Ruppelt-Lorz, Abiola Senok, Ali M. Somily, Edet E. Udo, Ralf Ehricht

**Affiliations:** 1 Leibniz Institute of Photonic Technology (IPHT), Jena, Germany; 2 InfectoGnostics Research Campus Jena, Jena, Germany; 3 Institute for Medical Microbiology and Hygiene, Medical Faculty “Carl Gustav Carus”, Technische Universität Dresden, Dresden, Germany; 4 Institute for Clinical Chemistry and Laboratory Medicine, Municipal Hospital Dresden, Dresden, Germany; 5 Centre National de Référence des Staphylocoques, Institut des Agents Infectieux, Hospices Civils de Lyon, Lyon, France; 6 Department of Microbiology, Faculty of Medicine, Kuwait University, Jabriya, Kuwait; 7 Department of Internal Medicine and Infectious Diseases, Faculty of Veterinary Medicine, Mansoura University, Mansoura, Egypt; 8 BLINK AG, Jena, Germany; 9 PTC-Phage Technology Center GmbH, Bönen, Germany; 10 Department of Hygiene and Zoonoses, Faculty of Veterinary Medicine, Mansoura University, Mansoura, Egypt; 11 Formerly Friedrich-Loeffler-Institut (Federal Research Institute for Animal Health), Institute of Bacterial Infections and Zoonoses, Jena, Germany; 12 College of Medicine, Mohammed Bin Rashid University of Medicine and Health Sciences, Dubai, United Arab Emirates; 13 Oral and Biomedical Sciences, School of Dentistry, Cardiff University, Cardiff, UK; 14 Department of Pathology and Laboratory Medicine, College of Medicine, King Saud University Medical City and King Saud University, Riyadh, Saudi Arabia; 15 Institute of Physical Chemistry, Friedrich Schiller University, Jena, Germany

**Keywords:** Microbiology, Molecular biology, Diseases

## Abstract

While many data on molecular epidemiology of MRSA are available for North America, Western Europe and Australia, much less is known on the distribution of MRSA clones elsewhere. Here, we describe a poorly known lineage from the Middle East, CC1153, to which several strains from humans and livestock belong. Isolates were characterised using DNA microarrays and one isolate from the United Arab Emirates was sequenced using Nanopore technology. CC1153 carries *agr* II and capsule type 5 genes. Enterotoxin genes are rarely present, but PVL is common. Associated *spa* types include t504, t903 and t13507. PVL-positive CC1153-MSSA were found in Egyptian cattle suffering from mastitis. It was also identified among humans with skin and soft tissue infections in Saudi Arabia, France and Germany. CC1153-MRSA were mainly observed in Arabian Gulf countries. Some isolates presented with a previously unknown SCC*mec*/SCC*fus* chimeric element in which a *mec* B complex was found together with the fusidic acid resistance gene *fusC* and accompanying genes including *ccrA/B-*1 recombinase genes. Other isolates carried SCC*mec* V elements that usually also included *fusC*. Distribution and emergence of CC1153-MRSA show the necessity of molecular characterization of MRSA that are resistant to fusidic acid. These strains pose a public health threat as they combine resistance to beta-lactams used in hospitals as well as to fusidic acid used in the community. Because of the high prevalence of *fusC*-positive MRSA in the Middle East, sequences and descriptions of SCC elements harbouring *fusC* and/or *mecA* are reviewed. When comparing *fusC* and its surrounding regions from the CC1153 strain to available published sequences, it became obvious that there are four *fusC* alleles and five distinct types of *fusC* gene complexes reminiscent to the *mec* complexes in SCC*mec* elements. Likewise, they are associated with different sets of *ccrA/B* recombinase genes and additional payload that might include entire *mec* complexes or SCC*mec* elements.

## Introduction

*Staphylococcus aureus* (*S. aureus*) is a common coloniser, or pathogen, among humans as well as among wild and domestic animals. It can cause a broad variety of infections that include not only superficial skin and soft tissue infections (SSTI) but also life-threatening conditions such as sepsis, infective endocarditis and pneumonia. While beta-lactams are crucial for treatment, resistant strains, so-called methicillin-resistant *Staphylococcus aureus* (MRSA), were first reported nearly 60 years ago^1^. Beta-lactam resistance in MRSA is caused by alternative penicillin-binding proteins encoded by different *mec* genes/alleles, out of which *mecA* is the most common and widespread one^2, 3^. The *mecA* gene is located on large and complex genetic elements, known as SCC*mec* (“staphylococcal cassette chromosome” or “staphylococcal chromosomal cassette” harbouring *mecA*) in which it is linked to *ccr* recombinase genes and, variably, to additional genes encoding antimicrobial or heavy metal resistance^4–11^. Originally, MRSA was restricted to healthcare settings, but from the mid-1990s on, infections with community-acquired MRSA (CA-MRSA) were observed. Many, but not all, CA-MRSA strains carry emerging SCC*mec* types IV or V, as well as Panton-Valentine leukocidin (PVL; encoded by *lukS/F*-PV genes). This is a cytotoxic, pore forming toxin localized on prophages. It is associated with recurrent, chronic and/or severe SSTI as well as with rapidly progressing necrotising pneumonia. The emergence and spread of PVL-positive CA-MRSA has extensively been studied in the United States and Australia, where they are common, as well as in Western Europe, where they pose a comparatively minor problem. Less data is available for other parts of the world, but during recent years it became obvious that PVL-positive CA-MRSA are an important public health issue in Mediterranean countries, the greater Middle East, Pakistan and India. The Arabian Gulf countries are of special interest because they are a major destination for migrants, expatriate workers, tourists and pilgrims from all over the world. This might result in importation, exchange and exportation of MRSA strains epidemic to other regions of the world. Indeed, in these countries, a high degree of diversity of MRSA strains has been observed with several strains being linked to other parts of the world^12–15^.

Since PVL is associated with clinically relevant skin conditions, topical treatments are frequently used. One option is fusidic acid, a steroid antibiotic known since the 1960s. Unfortunately, an excessive consumption of fusidic acid might quickly lead to an emergence of resistance, as it is well documented from New Zealand^16, 17^. Fusidic acid resistance is common in Middle Eastern/Arabian Gulf states, usually being due to plasmid-borne *fusB/far1*^13, 18–24^ or SCC-associated *fusC*^12, 25–32^.

Here, we describe a poorly known *S. aureus* lineage from the Middle East, CC1153, to which several distinct strains from humans and from livestock belong. Most isolates identified are PVL-positive, and many are MRSA that additionally harbour *fusC.* One CC1153 strain harboured a previously undescribed SCC*mec/fus* composite element. This observation prompted Nanopore sequencing and subsequent analysis of its genome. SCC*mec/fus* are reviewed and five distinct gene clusters associated with *fusC* are defined.

## Results

### Description of the clonal complex

CC1153 included sequence types (ST) 1153 (1–13–1–1–124–5–3) and ST2482 (1–141–1–1–124–8–3). Possible RIDOM *spa* types are t504 (26–17–20–17–12), t903 (26–22–19–17–17–20–17–12) and t13507 (26–22–19–17–17–20–17). Isolates belonged to *agr* group II and capsule type 5. The *sasG* gene was present but *cna* and the enterotoxin homologue ORF CM14 are uniformly absent. CC1153 isolates did not harbour *egc* locus and leukocidin genes *lukD/E* were variably present.

When analysing 154 core genomic markers (Supplement 3), CC1153 appeared to be most related to CC6, CC7 and CC1290. When comparing these 154 markers, all together consisting of 124.248 nucleotides, to CC6 strain PFESA1528, (GenBank FKTB), CC7 strain TCH959 (GenBank AASB) and CC1290 strain 015H (GenBank FMMV), differences of respectively, 0.38%, 0.37% and 0.38% were noted. As comparison, for CC1 (MW2, BA000033), CC5 (N315, BA000018), CC8 (TCH1516, CP000730) and *S. argenteus* CC1850 (MSHR1132, FR821777) differences were 0.45%, 0.51%, 0.40%, and 9.18%, respectively. However, in comparison to CC6, CC7 and CC1290, different *agr* groups (*agr* II in CC1153, *agr* group I in the others) and capsule types (*cap* 5 in CC1153, *cap* 8 in the others) as well as presence of *cna* in CC6 and absence of *sasG* from CC7 were noted. As these divergent loci are localised at distant positions across the genome, an emergence of CC1153 from these lineages by a single replacement of a fragment of chromosomal DNA appears to be unlikely.

### CC1153-MSSA strains

Twenty-three isolates of CC1153-MSSA were characterised (see Supplemental File 1). They originated from France (11/23), Egypt (6/23), Saudi Arabia (5/23) and Germany (1/23). The Egyptian isolates originated from cattle with subclinical mastitis, all other isolates were of human origin. CC1153-MSSA isolates, including those from cattle, are usually PVL-positive with *lukF/S*-PV being detected in 21 out of 23 isolates. Some isolates, mainly Saudi Arabian (n = 4) and French (n = 3) ones, harboured enterotoxin genes *sek* and *seq*. The staphylokinase gene *sak* and *scn* (staphylococcal complement inhibitor) were always present while only one isolate was positive for *chp* (chemotaxis-inhibiting protein). All isolates carried the penicillinase operon (*blaZ/blaI/blaR*) while other resistance genes were only sporadically found (*erm*(C), *msr*(A), *mph*(C), *aadD* and *fusB/far*1; each once in 23 isolates).

### CC1153-MRSA strains

Twenty-six isolates of CC1153-MRSA were identified and characterised (see Supplemental File 1).

Nine isolates were assigned to different variants of SCC*mec* V or VT elements. Five of these isolates came from Kuwait, one from Riyadh, Saudi Arabia, and two from the UAE (one each from Dubai and Umm-al-Quwain) and one from an Egyptian child living in Germany. All isolates, except the oldest one (isolated in 2009^33^) also carried the *fusC* gene. In two isolates, the SCC*mec/fusC* composite element was further characterised using a second microarray^9^ assigning them to SCC*mec* V + *fusC* (rather than to SCC*mec* VT + *fusC*). One isolate (from Kuwait) yielded the same pattern (with signals for *mecA, ugpQ, fusC, mvaS*-SCC, Q4LAG7, *ccrAA, ccrC,* SCCterm3, SCCterm10) as observed in a possibly livestock–(i.e., camel-) associated CC15 strain from Saudi Arabia^28, 34^. The other one (from Germany/Egypt) yielded signals for *mecA, ugpQ, fusC, mvaS*-SCC, Q4LAG7, *ccrAA, ccrC* and SCCterm11 possibly indicating a difference affecting the *SCCmec/orfX* junction site and/or another SCC*mec/fus* subtype. Seven of these isolates harboured PVL genes. Enterotoxin genes and *chp* were not identified, but *sak* and *scn* were always present. Eight out of the nine isolates harboured *fusC*. All were positive for *blaZ/blaI/blaR*. The gentamicin resistance gene *aacA-aphD* was found in eight isolates, the tetracycline resistance marker *tet*(K) in four isolates.

Seventeen CC1153-MRSA isolates belonged to a strain which, to the best of our knowledge, carried an unknown SCC*mec/fusC* chimeric element. Three of these isolates were investigated with the second array yielding signals with *mecA, ugpQ*, Delta *mecR*1, *fusC,* Q4LAG7 (MSSA476), *mvaS-*SCC, *ccrA*-*1*, *ccrB*-*1* and *dcs*. This prompted genome sequencing of one isolate, henceforth designated M58 (see below). Isolates with the new chimeric element originated from Kuwait (n = 14), UAE (n = 2) and France (n = 1). All but one were positive for *lukF/S*-PV genes. Genes *sak* and *scn* were always present while enterotoxin genes were not detectable. Sixteen isolates of this strain harboured *blaZ/blaI/blaR*, and *erm*(C) was found once.

### Description of the SCC*mec* element in M58

**Figure 1:**
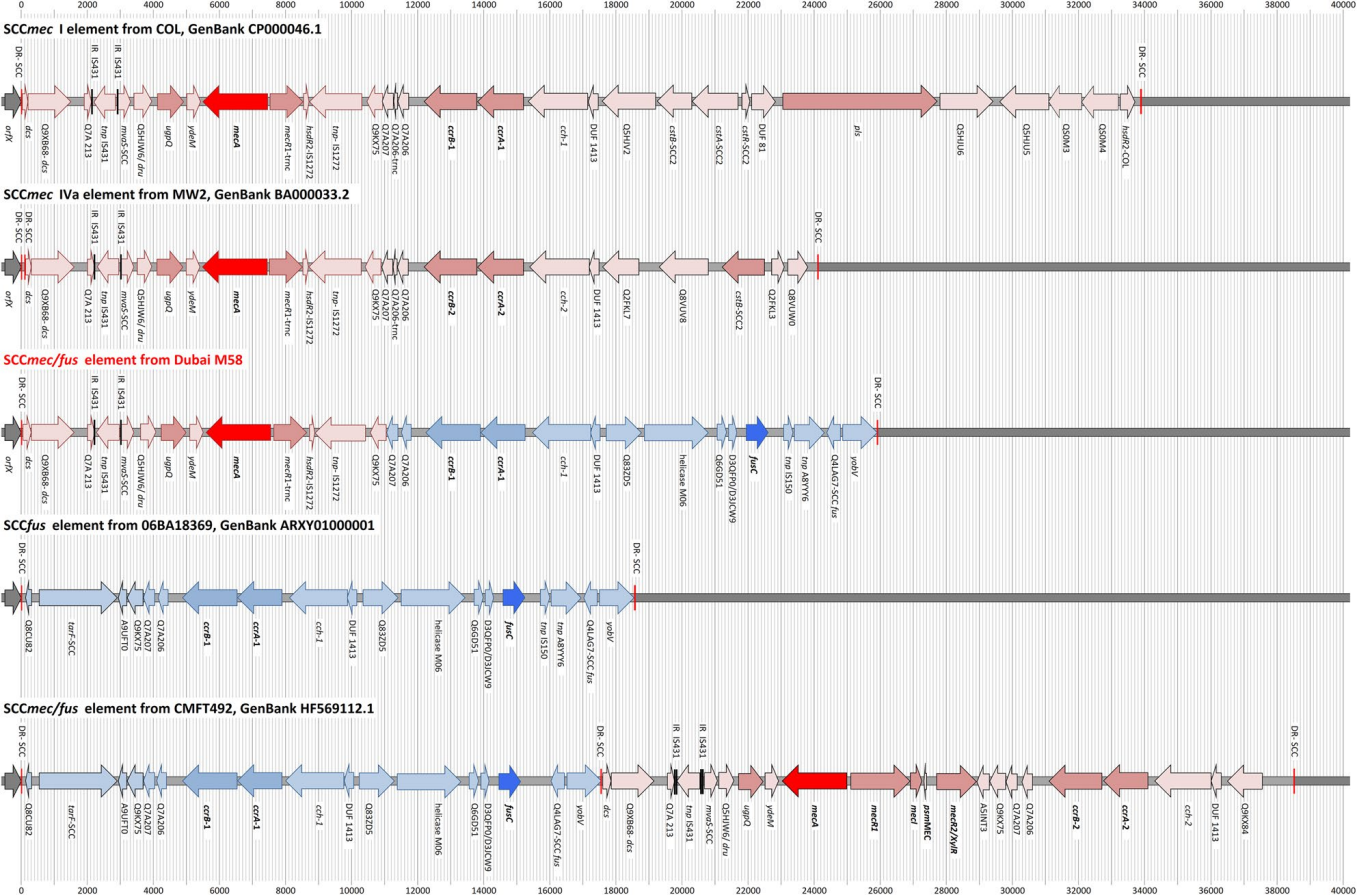
The SCC*mec/fus* element in M58 in comparison to some reference sequences (COL, MW2, 06BA18369 and CMFT492).

One of the seventeen isolates with an apparent unknown *SCCmec/fusC* chimeric element was subjected to genome sequencing (Nanopore) to characterise this element (see GenBank CP065857.1). Its gene content of the SCC*mec*/*fusC* element is summarised in Table 1, and Fig. 1 provides a graphical overview as well as a comparison to other, previously published, reference sequences.

**Table 1:** The SCC*mec*/SCC*fus* composite element in M58.

Gene ID	Explanation	Position in SCC (nt)	Position in genome (nt)	Length (nt)	Direction	Sequence identical to
*orfX*	(23S rRNA methyltransferase)		33,698–34,177	480	Forward	
sRNA6	(Antisense RNA associated with *orfX*)		33,878–34,161	284		
DR_SCC	Direct repeat of SCC	1–19	34,159–34,177	19		
*dcs*-L1	Downstream constant segment, locus 1	20–301	34,178–34,459	282		MW2, BA000033 [34169:34450]
Q9XB68-*dcs*	Putative protein	302–1596	34,460–35,754	1295	Forward	Strain 21172, AFEF01000013 [388744:390039]
Q7A213	Putative protein	2011–2250	36,169–36,408	240	Forward	COL, CP000046 [36082:36321]) and MW2, BA000033 [36161:36400])
IR_IS431	Inverted repeat of IS431	2223–2238	36,381–36,396	16		COL, CP000046 [36294:36309] and MW2, BA000033 [36373:36388]
tnpIS431	Transposase for IS431	2282–2956	36,440–37,114	675	Reverse (trunc.)	COL, CP000046 [36353:37027]
Teg143	Trans-encoded RNA associated with tnpIS431	2987–3020	37,145–37,178	34		COL, CP000046 [37058:37091] and MW2, BA000033 [37137:37170]
IR_IS431	Inverted repeat of IS431	2997–3012	37,155–37,170	16		COL, CP000046 [37058:37091] and MW2, BA000033 [37137:37170]
*mvaS*-SCC	Truncated 3-hydroxy-3-methylglutaryl CoA synthase	3029–3381	37,187–37,539	353	Forward	COL, CP000046 [37100:37452] and MW2, BA000033 [37179:37531]
Q5HJW6	Putative protein	3479–3709	37,637–37,867	231	Forward	COL, CP000046 [37550:37780] and MW2, BA000033 [37629:37859]
*dru*	SCC direct repeat units	3619–4056	37,777–38,214	438		COL, CP000046 [37690:38127]
*ugpQ*	Glycerophosphoryl diester phosphodiesterase	4258–5001	38,416–39,159	744	Forward	COL, CP000046 [38329:39072] and MW2, BA000033 [38288:39031]
*ydeM*	Putative dehydratase	5098–5526	39,256–39,684	429	Forward	COL, CP000046 [39169:39597] and MW2, BA000033 [39128:39556]
*txbi_mecA*	Bidirectional rho-independent terminator of *mecA*	5517–5581	39,675–39,739	65		
*mecA*	Penicillin binding protein 2a	5572–7578	39,730–41,736	2007	Reverse	MW2, BA000033.2 [39602:41608]
*mecR1*-trunc	Methicillin resistance operon repressor 1, truncated as in SCCmec I, IV, V	7678–8652	41,836–42,810	975	Forward (trunc.)	
*hsdR*2-IS1272	Fragment of type I restriction-modification system endonuclease	8653–8886	42,811–43,044	234	Truncated	COL, CP000046 [42724:42957] and MW2, BA000033 [42683:42916]
tnpIS1272	Transposase for IS1272	8887–10,410	43,045–44,568	1524	Reverse	COL, CP000046 [42958:44481] and MW2, BA000033 [42917:44440]
Q9KX75	Putative protein	10,546–11,052	44,704–45,210	507	Reverse	
Q7A207	Putative protein	11,068–11,379	45,226–45,537	312	Reverse	06BA18369, ARXY01000001 [134883:135194]
Q7A206	Putative protein	11,466–11,816	45,624–45,974	351	Reverse	06BA18369, ARXY01000001 [135281:135631]
*ccrB-1*	Cassette chromosome recombinase B, type 1	12,282–13,907	46,440–48,065	1626	Reverse	
*ccrA-1*	Cassette chromosome recombinase A, type 1	13,929–15,278	48,087–49,436	1350	Reverse	06BA18369, ARXY01000001 [137744:139093]
*cch-1*	Cassette chromosome helicase	15,466–17,235	49,624–51,393	1770	Reverse	CMFT532, HF569114 [8453:10222]
DUF1413-06BA18369	Putative protein associated with *cch*	17,235–17,525	51,393–51,683	291	Reverse	06BA18369, ARXY01000001 [141050:141340]
Q83ZD5	Putative protein	17,696–18,769	51,854–52,927	1074	Forward	06BA18369, ARXY01000001 [141511:142584]
Helicase M06	DEAD/DEAH box helicase domain protein	18,863–20,803	53,021–54,961	1941	Forward	06BA18369, ARXY01000001 [142678:144618]
Q6GD51	Putative protein	21,060–21,368	55,218–55,526	309	Forward	06BA18369, ARXY01000001 [144875:145183]
D3QFP0-SCC	Putative lipase/protease	21,415–21,653	55,573–55,811	239	Reverse (trunc.)	06BA18369, ARXY01000001 [145230:145468] and MSSA476, BX571857 [52281:52519]
D3JCW9	Putative protein	21,478–21,636	55,636–55,794	159	Forward	06BA18369, ARXY01000001 [145293:145451] and MSSA476, BX571857 [52344:52502]
*fusC*	Fusidic acid resistance protein C	21,954–22,592	56,112–56,750	639	Forward	06BA18369, ARXY01000001 [145769:146407] and MSSA476, BX571857 [52820:53458]
tnpIS150	Transposase of IS150	23,068–23,382	57,226–57,540	315	Forward	CMFT463, HF569110 [16043:16357]
tnp A8YYY6	Transposase	23,394–24,209	57,552–58,367	816	Forward (trunc.)	
Q4LAG7-SCC*fus*	Putative protein	24,356–24,784	58,514–58,942	429	Reverse	06BA18369, ARXY01000001 [148171:148599]
*yobV*	Transcriptional regulator	24,864–25,793	59,022–59,951	930	Forward	06BA18369, ARXY01000001 [148679:149608]
DR_SCC	Direct repeat of SCC	25,890–25,908	60,048–60,066	19

In short, the element comprised a *mec* complex B, *ccrA/B-1* recombinase genes and *fusC*, while *tirS* (that commonly accompanies *fusC*^35^) was absent. The gene *pls*-SCC, which normally is part of SCC*mec* I, was also absent. This constellation raises the question whether the element was derived from a SCC*mec* I element truncated by an insertion of *fusC,* or if it was a *mec* complex B element from a SCC*mec* I or IV element supplemented by *fusC* and accompanying *ccrA/B-1* recombinase genes.

The actual *mecA* allele was identical to one which is widespread in SCC*mec* IV strains including, for instance, MW2^2^ but differing from the one in COL. The *mec* complex B was followed by some genes encoding “putative proteins” and by *ccrB-1* and *ccrA-1* recombinase genes, as it was also the case in SCC*mec* I. A closer inspection of the sequence of the genes encoding “putative proteins” and of *ccrB-1* revealed differences compared to the corresponding sequences in SCC*mec* I. The *ccrB-1* allele from SCC*mec* I in COL differed by 7.2% of its nucleotides while *ccrA-1* was too conserved to allow a meaningful analysis.

This prompted a search for possible donors of recombinase and *fusC-*associated genes. While *fusC* itself was identical to MSSA Sanger 476, GenBank BX571857.1, the surrounding region was different in both gene content (most notably, in absence of *tirS*) as well as in gene sequences (8.4% difference in *ccrB-1*). Most closely related sequences of *fusC-*associated genes were identified in the *S. aureus* CC5 strain 06BA18369, GenBank ARXY, and in the *Staphylococcus hominis subsp. hominis* strain NTUH-3390, GenBank KY643657.1. These strains carry Q8CU82, *tarF-*SCC, A9UFT0, Q9KX75, Q7A207, Q7A206, *ccrB-1, ccrA-1, cch-1*, DUF1413, Q83ZD5, helicase M06, Q6GD51, D3QFP0-scc, D3JCW9, *fusC*, tnpIS150, tnp_A8YYY6, Q4LAG7-SCC*fus* and *yobV* (for explanations and GenBank entries of the genes discussed, see Table 1 and Supplemental File 4). However, Q8CU82, *tarF*-SCC-1 and A9UFT0 were absent in M58. The gene encoding the putative protein Q9KX75 in M58 was virtually identical to the ones in COL and MW2 but differed from the one in 06BA18369 and NTUH-3390, GenBank KY643657.1. From Q9KX75 on downstream, however, 06BA18369 and NTUH-3390 sequences were virtually identical to the ones in M58.

### The PVL prophage in M58

The sequence of the PVL prophage in the genome of M58 (CP065857.1) was identical to the one in the *S. aureus* CC1153 strain 3688STDY6124889, GenBank FQHT01000001.1. It was also identical to the PVL prophage in USA300-TCH1516, CP000730.1.

M58 was shown by a lateral flow assay to secrete detectable amounts of PVL. This was also the case for two other CC1153-MRSA-PseudoSCC*mec* [class B + *fus* + *ccrAB1*] isolates as well as for three of the CC1153-MSSA isolates.

## Discussion

### CC1153 and the SCC*mec*/*fusC* element in isolate M58

We describe a clonal complex of *S. aureus* that we identified in several Middle Eastern countries. A couple of publicly available genomes, deposited in GenBank (GenBank FQHT01000000) and/or the Short Read Archive (SAMEA2661948, SAMEA2661956, SAMEA2662240, SAMEA2662319, SAMEA2710354, SAMEA2710468, SAMEA3214613, SAMEA3448866, SAMEA3448996, SAMEA4547522, SAMN03289718) belong to it, but to the best of our knowledge, this clonal complex has not yet been reviewed. Three of these sequences originated from Thailand (GenBank FQHT01000000.1 as well as BioSamples SAMEA3448866 and SAMEA3448996), and one from the United Kingdom (SAMN03289718) while for the others, no locations were reported. An additional observation of CC1153 isolates originated from Myanmar^36^. Our isolates were collected in the greater Middle East (Egypt and Arabian Gulf countries) and Western Europe although at least one of the European cases had connections to Egypt. There are no data confirming or explaining a discontinuous distribution in the Middle East and in South-East Asia. However, the presence of millions of expatriate South-East Asians in the Gulf countries could easily explain a transmission of a *S. aureus* lineage into either direction.

An interesting observation is the presence of CC1153 in Egyptian bovines^37^. The detection of PVL (rather than of *lukM/lukF*-P83) and of haemolysin-beta-converting prophages in these isolates indicates a human provenance of these isolates so that the cattle probably served as sentinels for an unrecognised epidemiological situation among humans in the Nile Delta.

The majority of CC1153, including MRSA and MSSA, is PVL-positive harbouring the same prophage (in M58 and FQHT01000001.1) as other pandemic strains such as USA300.

CC1153-MRSA were mostly identified in Kuwait and the UAE. The clear majority, i.e. all isolates except the oldest one^33^, harboured SCC*mec*/SCC*fu*s chimeric elements and the most common variant that could be described either as SCC*mec* I + *fusC* element or as a pseudoSCC*mec* class B + *fusC* + *ccrA/B*-1 element was sequenced. Sequence analysis also revealed (see above and Table 1) that *ccrB*-*1* and accompanying genes are more related to alleles from other SCC*fus* elements rather than to the ones from SCC*mec*. Thus, a description as a pseudoSCC*mec* class B + *ccrA/B-1* + *fusC* element should be regarded as the correct one. The *mec* complex B could have been derived from either a SCC*mec* I or SCC*mec* IV element. However, the latter one was more probable based on of the MW2-like allele of *mecA*.

The entire region associated with *fusC* (encompassing Q7A207, Q7A206, *ccrB-1, ccrA-1*, *cch-*1, DUF1413, Q83ZD5, helicase M06, Q6GD51, D3QFP0-SCC, D3JCW9, *fusC,* tnpIS150, tnp_A8YYY6, Q4LAG7-SCC*fus* and *yobV*) could be seen as one mobile genetic element that got introduced into a CC1153-MRSA replacing Q7A207, Q7A206 and the *ccr* recombinase genes that previously belonged to its SCC*mec* element. This set of genes was also found in a *Staphylococcus hominis subsp. hominis* strain and a CC5-MSSA from Canada (06BA18369 GenBank ARXY00000000.1) as described above. Furthermore, MRSA strains from Saudi Arabia^25^ (as represented by isolates CMFT492, HF569112.1 and CMFT532, GenBank HF569114.1; see Tables 2, 3, 4 and 5) also carried the same region associated with *fusC* (differing from the one in M58 only in minor random deletions) as part of complex chimeric SCC*mec* II elements. In CMFT492, this cluster was inserted between *orfX* and a truncated SCC*mec* II element that lacked the *kdp* locus, *cstA/B/R* and the transposons introducing *ble/aadD* and *erm*(A)/*ant*9. In CMFT532 and other strains (see Tables 2, 3, 4 and 5), the region associated with *fusC* was inserted between *orfX a*nd a normal SCC*mec* II element. In these strains, additional markers (sccterm13, Q8CU82, *tarF*-SCC, A9UFT0, Q9KX75) were also associated with the *fusC* element that were absent in M58. Furthermore, there were also strains such as FORC 090, GenBank CP029198.1 or AR466, CP029080.1 in which *fusC* and its immediate neighbours (Q83ZD5, helicase M06, Q6GD51, D3QFP0-SCC, D3JCW9, tnpIS150, tnp A8YYY6, Q4LAG7-SCC*fus* and *yobV*) were accompanied by other *ccr* recombinase genes and other genes upstream, towards *orfX.* This prompted us to review published sequences and to compare them with the CC1153 strain described herein to sort and to classify the different gene clusters accompanying *fusC.*

**Table 2:**
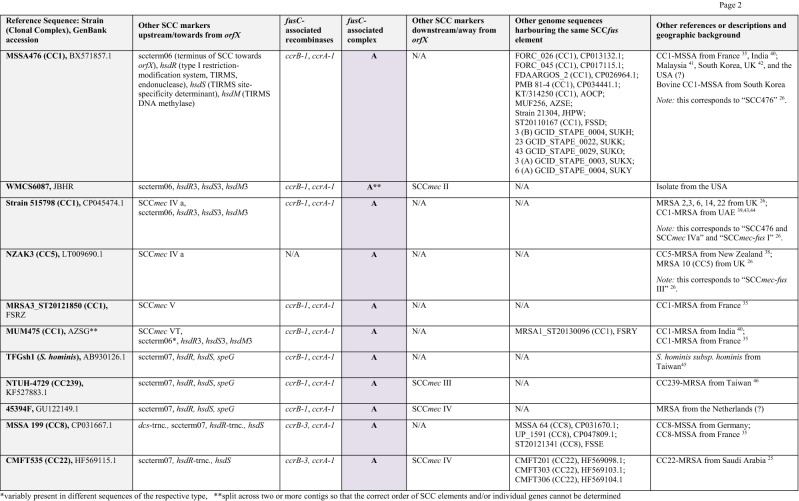
Staphylococcal Cassette Chromosomes with a *fusC*-associated complex **A** consisting of Q6GD54, Q6GD5, *tirS*, Q6GD51, D3QFP0-SCC, D3JCW9, *fusC*, sccterm03, Q6GD49, Q8CU43, Q4LAG7-SCC*fus* and *yobV.* Please note that this is an abridged version (with genes encoding “putative proteins” omitted); for a more detailed version, see Supplement 5.

References^25, 26, 35, 38–46^.

*Variably present in different sequences of the respective type.

**Split across two or more contigs so that the correct order of SCC elements and/or individual genes cannot be determined.

**Table 3:**
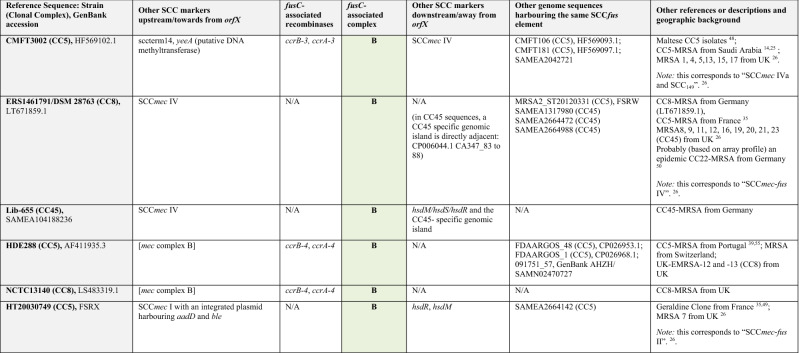
Staphylococcal Cassette Chromosomes with a *fusC*-associated complex **B** consisting of Q6GD54, Q6GD53, *tirS*, Q6GD51, D3QFP0-SCC, D3JCW9 and *fusC* (14A > C; 150 T > G; 290G > C; 537A > T; 632 T > C). Please note that this is an abridged version; for a more detailed version, see Supplement 5.

References^14, 25, 26, 35, 47–51^.

**Table 4:**
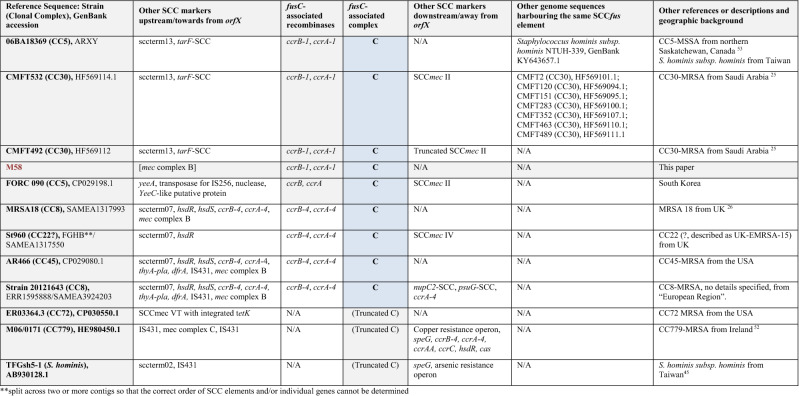
Staphylococcal Cassette Chromosomes with a *fusC*-associated complex **C** consisting of Q83ZD5, helicase M06, Q6GD51, D3QFP0-SCC, D3JCW9, *fusC*, tnpIS150, tnp A8YYY6, Q4LAG7-SCC*fus* and *yobV*. Please note that this is an abridged version; for a more detailed version, see Supplement 5.

References^25, 26, 45, 52, 53^.

**Split across two or more contigs so that the correct order of SCC elements and/or individual genes cannot be determined.

**Table 5:**
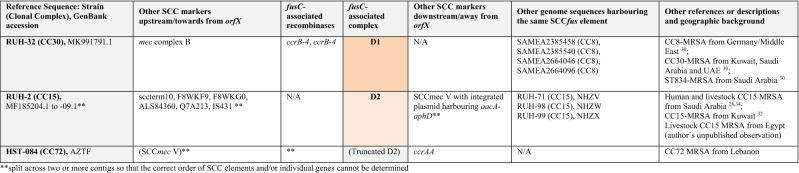
Staphylococcal Cassette Chromosomes with a *fusC*-associated complex **D** consisting of Helicase M06, Q6GD51, D3QFP0-SCC, D3JCW9, *fusC* (309G > A), sccterm03, Q6GD49, Q8CU43, Q4LAG7-SCC*fus* and *yobV* (**D1**) or HelicaseM06, Q6GD51, D3QFP0-SCC, D3JCW9, *fusC* (486 T > C), sccterm03, Q6GD49, and Q8CU43 (**D2**). Please note that this is an abridged version; for a more detailed version, see Supplement 5.

References^28, 30, 32, 34^.

**Split across two or more contigs so that the correct order of SCC elements and/or individual genes cannot be determined.

### Review of *fusC* elements

**Figure 2:**
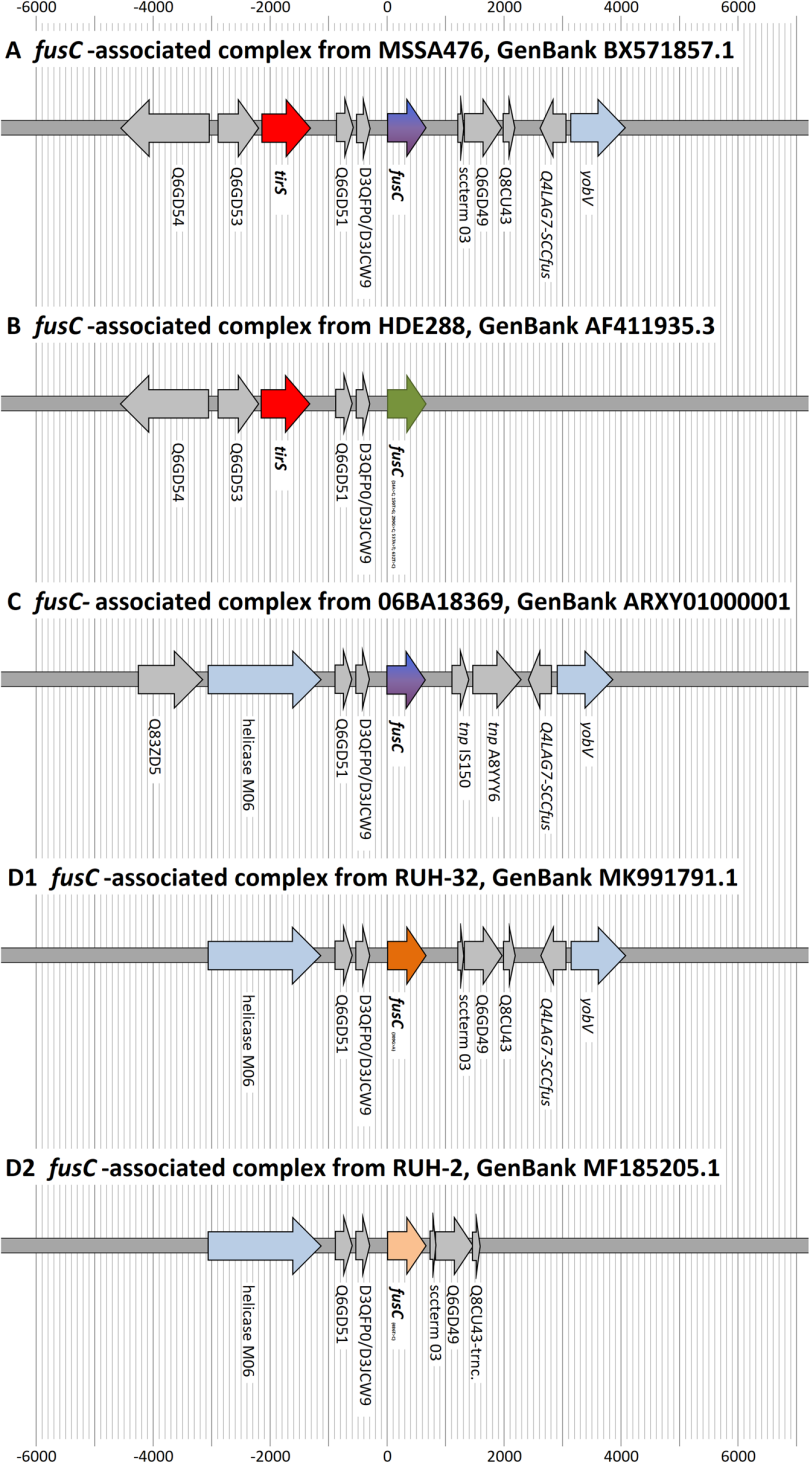
The different sets of genes directly accompanying *fusC* in M58 and in previously published SCC*fus* or SCC*mec*/*fus* elements.

When comparing the region around *fusC* from M58 to published sequences, it became obvious that 64 published *S. aureus* sequences (plus three S*. hominis* sequences and nine un-assembled *S. aureus* sequences from the Short Read Archive) cluster into 31 different SCC*fus* or SCC*mec/fusC* chimeric or composite elements (Tables 2, 3, 4, 5/Supplemental File 5). None of these fully matched the M58 sequence. When considering only the immediate region around *fusC*, strains were identified in which the same gene cluster as M58 was present and it was observed that there are only four, possibly five, different sets of genes directly accompanying this resistance gene (Tables 2, 3, 4, 5/Supplemental File 5 and Fig. 2).

These sets could be regarded as fixed gene complexes in analogy to the *mec* complexes A (in SCC*mec* II and III), B (in SCC*mec* I and IV) and C (in SCC*mec* V). Likewise, they are also associated with different sets of SCC-recombinase genes including alleles of *ccrA-1/ccrB-1, ccrA-1/ccrB-3, ccrA-3/ccrB-3, ccrA-4/ccrB-4, ccrA/ccrB1* (FORC_90) and *ccrAA/ccrC.* Resulting “SCC*fus*” elements can, besides *fusC-*complexes and recombinase genes, also carry additional payload including *tarF* (teichoic acid biosynthesis protein F)*, speG* (spermidine N-acetyltransferase), various variants of type I restriction-modification systems or *mec* complexes. They also can be linked to entire SCC*mec* elements resulting in complex genomic islands sometimes even including multiple sets of recombinase genes. These additional components can be localized upstream (towards *orfX*) or downstream (see Tables 2, 3, 4, 5/Supplemental File 5).

The longest known of the *fusC-*complexes (“**A**”, Table 2, see also Supplemental File 5 and Fig. 2), as in MSSA476, BX571857.1, comprises Q6GD54 (putative protein), Q6GD53 (putative protein), *tirS* (staphylococcal Toll/interleukin-1 receptor domain mimic), Q6GD51, D3QFP0-SCC, D3JCW9, *fusC*, sccterm03, Q6GD49 (putative protein), Q8CU43 (putative protein), Q4LAG7-SCC*fus* and *yobV* (for explanations and GenBank entries of the genes discussed, see Supplemental File 4). It can be found in MSSA, such as the prototypical CC1-MSSA sequence Sanger MSSA476, as well as in MRSA. It appears in MRSA strains (CC1 and CC5) with SCC*mec* IV elements, mainly form the Middle East^25, 28, 30, 31^, Australia and New Zealand^38^ as well as in SCC*mec* V strains from the Middle East.

A second *fusC-*complex (“**B**”, Table 3, see also Supplemental File 5 and Fig. 2) comprises Q6GD54, Q6GD53, *tirS*, Q6GD51, D3QFP0-SCC, D3JCW9 and *fusC.* Besides gene content, it also differs from all others in five characteristic single nucleotide polymorphisms (SNP) within the *fusC* gene (14A > C; 150T > G; 290G > C; 537A > T; 632T > C). This complex has apparently not yet been observed in MSSA but there are several MRSA strains harbouring it connected to various SCC*mec* elements. One is HDE288, as prototypical sequence the “New Paediatric” CC5-MRSA strain from Portugal^47^. Here, the *fusC-*complex is accompanied by *ccrA/B-4* genes and a *mec* complex B, a combination also referred to as SCC*mec* VI. Another CC5 (ST149) strain, known from Malta^48^, the Middle East^14, 25^ and UK^26^ harbours the same *fusC-*complex, together with *ccrA/B-*3 alleles and a SCC*mec* IV element. It also appears, although Q6GD54 is absent, in a SCC*mec* I MRSA strain from France (CC5, “Geraldine Clone”^49^).

A third *fusC-*complex (“**C**”, Table 4, see also Supplemental File 5 and Fig. 2) consisting of Q83ZD5, helicase M06, Q6GD51, D3QFP0-SCC, D3JCW9, *fusC*, tnpIS150, tnp A8YYY6, Q4LAG7-SCC*fus* and *yobV.* The *fusC* sequence is identical to the one in MSSA476, BX571857.1. This is the variant found in M58 and the other strains discussed above. In these strains, it is accompanied by a largely identical set of recombinase-associated genes. The carriage of sccterm13, Q8CU82, *tarF*-SCC, A9UFT0, Q9KX75 as additional payload is variable; in M58 these genes are replaced by the *mec* complex, while in the CMFT492, CMFT535 etc., the SCC*mec* element is located downstream away from *orfX*^25^. Strains FORC090 (CP029198.1), AR466 (CP029080.1), MRSA18 (SAMEA1317993)^26^ and 20121643 (ERR1595888/SAMEA3924203) harbour the same *fusC-*complex but in these sequences, it is accompanied by other recombinase alleles.

A fourth *fusC-*complex (“**D1**”, Table 5, see also Supplemental File 5 and Fig. 2) consists of Helicase M06, Q6GD51, D3QFP0-SCC, D3JCW9, *fusC*, sccterm03, Q6GD49, Q8CU43, Q4LAG7S-SCC*fus* and *yobV*. Its *fusC* gene has one characteristic SNP (309G > A). It was not yet found in MSSA, but in MRSA belonging to CC8, CC30 and ST834^30^. In these strains, it is accompanied by a *mec* complex B and a set of *ccrA/B-4* genes. It has been found neither in any other context, nor in MSSA strains.

A CC15 SCC*mec* V MRSA strain harbours a *fusC-*complex consisting of helicase M06, Q6GD51, D3QFP0-SCC, D3JCW9, *fusC*, sccterm03, Q6GD49 and Q8CU43. The *fusC* sequences contain a specific SNP (486T > C), but otherwise, gene content (apart from the lack of Q4LAG7-SCC*fus* and *yobV*) and the order of genes are the same as in the fourth complex (hence, “**D2**”, Table 5, see also Supplemental File 5 and Fig. 2). The *fusC-*complex itself is in all these sequences localised on one contig, but other associated markers such as SCC*mec*, *orfX* etc. are split across several contigs. One ST72 sequence (HST-084, AZTF00000000.1) has a SCC*mec*V/SCC*fus* chimeric element harbouring Helicase M06, Q6GD51, D3QFP0-SCC, D3JCW9 and a *fusC* gene with the same (486T > C) SNP but unfortunately, it is fragmented across several contigs. Another strain (ER03364.3, CP030550.1) with a SCC*mec*VT/SCC*fus* chimeric element also harbours helicase M06, Q6GD51, D3QFP0-SCC, D3JCW9 and *fusC*. However, the identity of *fusC* with the one in MSSA476, BX571857.1 (i.e., the absence of the 309G > ASNP) suggests it to be derived from the third *fusC-*complex (Table 4, see also Supplemental File 5)*.*

Further investigations on *fusC-*complexes associated with SCC*mec* V/VT are warranted as such isolates from diverse clonal complexes including CC5, CC97, CC121 and CC1153 (see above) have been observed, especially in the Arabian Gulf region.

Finally, truncated *fusC-*complexes were also observed as part of a very complex composite SCC*mec* element in a CC779 isolate M06/0171, HE980450.1^52^ and of a composite “pseudo-SCC” element (*i.e*., without *ccr* genes) in *Staphylococcus hominis subsp. hominis* TFGsh5-1, AB930128.1. As the third and the fourth complex, it encompasses genes for helicase M06, Q6GD51, D3QFP0-SCC, D3JCW9 and *fusC;* but its *fusC* allele indicates relation to the third one (Table 4, see also Supplemental File 5)*.*

There was no obvious phenotypical correlation of *fusC-*complexes to fusidic acid MICs, all tested strains (Supplemental File 6) were highly resistant regardless of their actual type of *fusC-*complex.

Further open questions are the timeframe of the evolution of SCC*fus* elements as well as their geographical origins. A wide variety of *fusC-*positive strains has not yet been sequenced. It would be interesting to know whether additional elements exist, and whether there are MSSA strains harbouring those *fusC-*complexes yet observed in MRSA only. The origin and evolutionary history of *tirS* is another open question. This virulence factor^35^ is present in two out of five *fusC-*complexes but to the best of our knowledge, it has never been observed in another context.

As previously discussed^30^, *fus*C was detected in as much as twenty-two different clonal complexes of *S. aureus,* CC1, CC5, CC6, CC7, CC8, CC15, CC22, CC30, CC45 [*agr* I], CC45 [*agr* IV], CC50, CC59, ST72, CC88, CC97, CC121, CC152, CC361, CC779, ST834, CC913 and CC1153 from essentially all parts of the world. This and the emergence of *fus*C-MRSA especially in the Middle East indicate a selective advantage associated with its presence. Fusidic acid can be administered intravenously, but this is not commonly done, and the intravenous formulation is not available everywhere. It is also used topically, as ointment for presumably staphylococcal skin conditions. Observations from New Zealand suggest a quick emergence of *fus*C-positive *S. aureus* in parallel to an increasing use of this compound^16, 38^. A co-evolution of SCC*mec* and SCC*fus* elements might result in a public health hazard, as strains with composite or chimeric elements are selected for both, in the hospital by beta-lactam administration as well as in the community by topical use of fusidic acid. Thus, antibiotic stewardship and infection control measures targeting MRSA in the hospital must be accompanied by restrictions to an uncontrolled over-the-counter sale of fusidic acid in outpatient settings as well as by a prudent use in outpatient settings.

## Materials and methods

### Isolates

A list of the isolates studied is provided in Supplement 1. Isolates were selected out of various typing and epidemiological projects based on array hybridisation profiles indicative for an affiliation to CC1153.

The CC1153-MRSA from the UAE were isolated from skin and wound infections. The CC1153-MRSA from Saudi Arabia also originated from a wound infection. One CC1153-MSSA from Saudi Arabia was a nasal colonizer from a healthcare worker, the others originated from skin and soft tissue infections (with three of them being identified during an earlier study^54^). The first CC1153-MRSA reported in Kuwait was cultured from a wound inflicted by a dog bite in 2009^33^. The other isolates were obtained between 2017 and 2020 mostly from wound infections of patients located in six hospitals. One isolate was isolated from a gynaecological swab, one from a nasal swab and one was obtained from blood culture. Egyptian isolates were identified from rural smallholder dairy cattle that showed sub-clinical mastitis, i.e., somatic cell counts > 200,000 cell/mL and positive results of California Mastitis Test. The isolates were collected from six different cows in a herd, consisting of 25 crossbred dairy cows, located at Dakahlia Governorate in the northeast of Cairo, Egypt. The milking procedure was performed manually in the examined cows, while the medical records of the farm revealed the usage of a wide spectrum of antibacterial agents^37^. French isolates (11 MSSA, 1 MRSA) had been isolated during infections (cutaneous (n = 8), respiratory (n = 3), blood culture (n = 1)) in ten different hospitals between 2010 and 2017. One German MRSA isolate was cultured from an abscess of an approximately half year-old Egyptian child whose family lives in Germany. One German MSSA isolate originated from an abscess.

PVL detection was performed on six isolates by an experimental lateral flow test^54^. Fusidic acid MICs were determined by agar gradient dilution tests with commercially available strips (01B10122 Fusidinsäure MIC Test Strip 0.016—256 µg/mL, Bestbion dx GmbH, Cologne, Germany).

### Microarray-based molecular characterization

Genotyping of all strains was performed using the *S. aureus* Genotyping Kit 2.0 system (Abbott [Alere Technologies GmbH, Jena, Germany]) microarray-based assay. The array covers 333 different targets related to approximately 170 different genes and their allelic variants. The list of target genes as well as sequences of probes and primers have previously been published along all relevant protocols^9, 39, 55^.

*Staphylococcus aureus* was cultivated on Colombia blood agar. The DNA extraction was performed using lytic enzymes (lysostaphin, lysozyme, RNAse) and buffer from the *S. aureus* Genotyping Kit 2.0 kit and Qiagen DNA extraction columns (Qiagen, Hilden, Germany) according to manufacturers’ instructions. Then, a linear amplification was performed using one primer for each target sequence. During the linear multiplex-amplification, biotin-16-dUTP was incorporated into the amplicons, which were then stringently hybridised to the specific probes on the microarray. After washing steps, hybridisation was detected using streptavidin horseradish peroxidase that triggered local precipitation at those spots where amplicon was bound. Microarrays were photographed and analysed with a designated reader and software (IconoClust, Abbott [Alere Technologies]). Analysis allowed detecting presence or absence of certain genes or alleles, as well as assignment to the clonal complex, strains, and SCC*mec* types.

### Whole-genome sequencing

Genomic DNA was isolated from an overnight culture grown at 37 °C on Columbia blood agar using a Macherey and Nagel NucleoSpin Microbial DNA kit (MACHEREY–NAGEL GmbH & Co. KG, Dueren, Germany).

The Nanopore Oxford MinION platform was used for sequencing the whole genome of the CC1153 isolate M58 from the UAE. Briefly, size selection was performed using AMPure beads in a ratio 1:1 (v/v) with the DNA sample. The DNA library was generated using the nanopore sequencing kit SQK-LSK109 and the native barcoding expansion kit EXP-NBD103 (Oxford Nanopore Technologies, Oxford, UK) according to manufacturer’s instructions. The used flowcell FLO-MIN106 (R9-Version) was primed by the flow cell priming kit EXP-FLP001 (Oxford Nanopore, Oxford, UK). The protocol named “1D Native barcoding genomic DNA” was used in version NBE_9065_v109_revB_23May2018 (Last update: 03/09/2018). The guppy basecaller (v4.4.2., Oxford Nanopore Technologies, Oxford, UK) translated and trimmed the MinION raw data (fast5) into quality tagged sequence reads (4000 reads per fastq-file). Flye (v2.8.1) was used to assemble all reads to one large contig. Then, a racon-medaka (racon v1.4.3; medaka v1.2.0) pipeline was applied for polishing (with settings and descriptions being provided as Supplement 7).

The genome sequence is provided under GenBank accession number CP065857.1.

### Phylogenetic analysis

A panel of 154 non-motile, core genomic markers was selected. Inclusion criteria were presence in all *S. aureus* clonal complexes analysed as well as uniform length in all genomes. The used genes as well as the genome sequences considered are listed in Supplemental File 3. Sequences were concatenated and analysed using SplitsTree 4.0^56^ using default settings (Supplemental File 3).

## Supplementary Information

Supplementary Information.
